# MicroRNA-1275 inhibits cell migration and invasion in gastric cancer by regulating vimentin and E-cadherin via JAZF1

**DOI:** 10.1186/s12885-019-5929-1

**Published:** 2019-07-29

**Authors:** Jia-Wei Mei, Zi-Yi Yang, Hong-Gang Xiang, Runfa Bao, Yuan-Yuan Ye, Tai Ren, Xue-Feng Wang, Yi-Jun Shu

**Affiliations:** 1 Department of General Surgery and Laboratory of General Surgery, Xinhua Hospital, Affiliated with Shanghai Jiao Tong University, School of Medicine, No. 1665 Kongjiang Road, Shanghai, 200092 China; 2 Shanghai Key Laboratory of Biliary Tract Disease Research, No. 1665 Kongjiang Road, Shanghai, 200092 China; 3 Institute of Biliary Tract Disease, Shanghai Jiao Tong University School of Medicine, No. 1665 Kongjiang Road, Shanghai, 200092 China; 4 Department of General Surgery, Pudong New Area People’s Hospital affiliated to Shanghai University of Medicine and Health Science, No. 490, South Chuanhuan Road, Pudong New Area, Shanghai, 201299 China

**Keywords:** Gastric cancer, miR-1275, JAZF1, Tumour progression, RNA interference

## Abstract

**Background:**

Emerging evidence has shown that miR-1275 plays a critical role in tumour metastasis and the progression of various types of cancer. In this study, we analysed the role and mechanism of miR-1275 in the progression and prognosis of gastric cancer (GC).

**Methods:**

Target genes of miR-1275 were identified and verified by luciferase assay and Western blotting. The function of miR-1275 in invasion and metastasis was analysed in vitro and in vivo in nude mice. The signal pathway regulated by miR-1275 was examined by qRT-PCR, Western blotting and chromatin immunoprecipitation analyses. The expression of miR-1275and JAZF1 were measured in specimens of GC and adjacent non cancerous tissues.

**Results:**

We identified JAZF1 as a direct miR-1275 target. miR-1275 supresses migration and invasion of GC cells in vitro and in vivo*,* which was restored by JAZF1 overexpression. Moreover, JAZF1 was recognized as a direct regulator of Vimentin. Knocking-down miR-1275 or overexpressing JAZF1 resulted in upregulation of Vimentin but downregulation of E-cadherin. Meanwhile, we validated in 120 GC patients specimens that low miR-1275expression and high JAZF1 mRNA expression levels were closely associated with lymph node metastasis and poor prognosis. The expression of JAZF1 in protein level displayed the correlations with Vimentin but inversely with E-cadherin.

**Conclusions:**

Increased miR-1275 expression inhibited GC metastasis by regulating vimentin/E-cadherin via direct suppression of JAZF1expression, suggesting that miR-1275 is a tumour-suppressor miRNA with the potential as a prognostic biomarker or therapeutic target in GC.

**Electronic supplementary material:**

The online version of this article (10.1186/s12885-019-5929-1) contains supplementary material, which is available to authorized users.

## Background

Gastric cancer (GC) is the fifth most common malignancy and third leading cause of cancer-related death throughout the world. The majority of patients with GC are diagnosed at an advanced stage, and their survival rate remains low due to the high probability of metastasis and recurrence [1, 2]. To improve the development of novel diagnostic and molecular treatment strategies for GC, an in-depth understanding of the molecular mechanisms of this disease is required [3–5]. It is of clinical importance to identify genes that contribute to the development of GC and have predictive value for its diagnosis or prognosis [6–8]. Despite extensive efforts to develop GC biomarkers, only modest success has been achieved in the biomarker-assisted diagnosis and treatment of GC [3, 9].

Metastasis, which includes a series of biological processes, is a crucial impediment to the effective treatment of GC patients [10]. Although many novel targeted molecules and the underlying mechanisms related to tumour cell motility and metastasis have been reported, metastasis of tumour is still hard to be fully explained. MicroRNAs (miRNAs), which function as endogenous regulatory RNA molecules, modulate protein expression by interacting with the 3′-untranslated regions (3′-UTRs) of target genes to downregulate their translation. Since the first miRNA was reported by Ambros and Ruvkun in 1993 [11], growing evidence has shown that miRNAs can function as either oncogenes or tumour suppressors in various cancers [12–14]. In our previous work, we demonstrated that miR-29c expression was frequently lower in gallbladder cancer (GBC) tissues than in adjacent nontumour tissues [15], indicating that miR-29c work as a potential tumour suppressor in GBC. In recent years, much attention has been attracted by the critical function of miRNAs in metastasis, but it is still need to be revealed that the significance of the majority of miRNAs to GC and its pathological relevance.

Juxtaposed with another zinc finger protein 1 (JAZF1) is a nuclear protein with three cysteine-2 histidine-2 (Cys2-His 2) zinc finger motifs [16]. JAZF1 could interact with testicular receptor 4 (TR4) to repress its transcriptional activity [17]. TR4 could function in weight gain and body fat accumulation by promoting the transcription of phosphoenolpyruvate carboxykinase (PEPCK) to activate gluconeogenesis [18, 19]. Additionally, it has been reported that JAZF1 functions in tumour progression in endometrial stromal sarcoma and prostate cancer [20–22]. in endometrial stromal tumours, A JAZF1-SUZ12 fusion protein disturbs chromatin formation by inhibiting polycomb repressive complex 2 (PRC2) complexes [23]. JAZF1 is also found playing an important role in prostate cancer and diabetes [21, 23, 24]; however, the molecular mechanism by which JAZF1 acts in these diseases has not yet been clarified.

In this study, we identified miR-1275 as a novel metastasis-inhibiting factor through targeting JAZF1, and miR-1275 overexpression could repress the metastasis and invasion of GC cells in vitro and in vivo. Furthermore, we identified a mechanism that JAZF1 directly regulated the expression of vimentin and E-cadherin by bindinig to their promoter regions in GC cells. Collectively, our study demonstrated a novel molecular link between low miR-1275 expression and cancer metastasis in GC tumorigenesis by downregulating vimentin and upregulating E-cadherin upregulation via JAZF1 targeting.

## Methods

### Patient specimens

Fresh GC tissue samples, tumour tissues and adjacent nontumor tissues (NATs) were collected from 120 patients with pathologically confirmed GC who underwent radical tumour resection in the Department of General Surgery, Xinhua Hospital from 2010 to 2013. All diagnoses of GC and lymph node metastasis were histologically examined. Overall survival (OS) was defined as the interval between the date of surgery and the date of death. Individuals with OS exceeding 36 months were censored. Fresh GC tissue samples were processed within 15 min of surgical removal, frozen and stored at − 80 °C until further use. The clinical and pathological features of these patients are described in Additional file 1: Table S1.

### Cell lines and cell culture

The human GC cell lines MGC803 (CRL-1739) and SGC-7901 (CRL-5822) were purchased from the Shanghai Institute for Biological Science, Chinese Academy of Sciences (Shanghai, China). The cell line was used in fewer than 6 months after receipt or resuscitation. Shanghai Institute for Biological Science performs anthentication of cell line via short tandem repeat profiling, karyotyping, and cyto c oxidase subunit I testing. Only mycoplasma tests were performed for the cell line authentication in our laboratory. We did not carry out additional testing to authenticate cell line, but its morphology and behaviour were consistent with ATCC descriptions. MGC803 cells were maintained in high-glucose DMEM (Gibco, NY, USA). SGC-7901 cells were cultured in Roswell Park Memorial Institute (RPMI) 1640 medium. All of the above media were supplemented with 10% foetal bovine serum (FBS) (Gibco) All cells were cultured at 37 °C in a humidified atmosphere comprising 95% air and 5% CO_2_.

### RNA isolation and quantitative real-time PCR (qRT-PCR)

Total RNA from tumours and cell lines was isolated with TRIzol reagent (Invitrogen) and converted into cDNA following the manufacturer’s instructions (Takara, Dalian, China). The PCR amplifications were performed with a StepOne™ Real-Time PCR System (Applied Biosystems, Foster City, USA) using SYBR® Green Real-Time PCR (Takara, Dalian, China) with GAPDH as an internal control. For miRNA quantification, cDNA was synthesized from total RNA with a Mir-X miRNA First-Strand Synthesis Kit (Clontech Laboratories, Inc.) and quantified via qPCR using a Mir-X miRNA qRT-PCR SYBR Kit (Clontech Laboratories, Inc.) with U6 as an internal control. The relative RNA expression levels were calculated using the comparative Ct method. The following primers were used in the qRT-PCR experiments: miR-1275 (AB Assay ID 002840); *U6* (AB Assay ID 001093); GADPH (AB Assay ID Hs00266705_g1); and JAZF1, sense, 5′-GGAGTCGGACAGCGATGATGAGT-3′, and antisense, 5′-GCTTCTCTTCCCCTCCATTCA-3′.

### Plasmids, RNA oligonucleotides, and target cell infection

#### JAZF1 3’UTR luciferase reporter assay

The full-length wild-type (WT) JAZF1 3’UTR sequence containing putative miR-1275 binding sites or mutant (Mut) JAZF1 containing miR-1275 mutant binding sites were synthesized by Shanghai Generay Biotech Co., Ltd. The DNA fragments were digested with Xbal and BamHI and cloned into the Xbal and BamHI sites of the pGL3 luciferase reporter plasmid (Promega, Madison, WI, USA).

#### Vector construction

The vimentin proximal promoter (− 1416/+ 72) was amplified from human genomic DNA by PCR. The (− 1416/+ 72) vimentin-Luc reporter plasmid was constructed from the luciferase reporter plasmid pGL3 with the digestion of KpnI and HindIII.

Anti-miR-1275, miR-1275 mimics, JAZF1 short interfering RNA (siRNA) (siRNA-1: 5′-UCUGUGACCAUUCUUAGCGUG-3′, siRNA-2: 5′-UUCACAUUCUUGUAUCUUU-3′), and their corresponding control RNAs were purchased from Biotend (Shanghai, China). For RNA oligonucleotide transfection, 50 nmol/L siRNA, 50 nmol/L anti-miR-1275, and 10 nmol/L miR-1275 mimic were used as indicated.

#### Lentiviral packaging and transduction

For the in vivo assay, miR-1275 and scramble miRNA were synthesized and inserted into the pFH1UGW lentiviral vector containing a eGFP reporter gene. Recombinant lentiviruses expressing JAZF1-siRNA or negative control were produced by GeneChem (Shanghai, China). The RNA and protein expression levels of JAZF1 were determined by qRT-PCR and Western blot assays. Cells were transfected using Lipofectamine 2000 (Invitrogen) according to the manufacturer’s instructions. (The nucleotides aboved can be found in Additional File 5)

### Luciferase reporter assay

For the relative luciferase reporter assay, cells (1 × 10^4^) were co-transfected with 500 ng of WT or Mut JAZF1 3’UTR. Each sample was co-transfected with 50 ng of the pRL-TK plasmid expressing Renilla luciferase to monitor the transfection efficiency. Relative luciferase and Renilla signals were calculated 48 h after co-transfection using a Dual Luciferase Reporter Assay Kit (Promega) according to the manufacturer’s procedure. Firefly luciferase activity was normalized to Renilla luciferase activity.

### In vitro tumorigenesis assays

Cell growth was determined by the Cell Counting Kit 8 (CCK-8, Dojindo) assay at 1, 2, 3, 4 and 5 days following transfection of MGC803 and NOZ cells. Anchorage-independent growth was assessed by the colony formation assay. Treated cells were seeded in a six-well culture plate (800 cells/well) and cultured for approximately 14 days.

### In vitro migration and invasion assays

For the in vitro wound healing assay, cells were seeded in 6-well plates, grown to 90% confluence, and then serum-starved for 24 h. MGC-803 (3 × 10^4^) and SGC-996 (4 × 10^4^) cells were then seeded into the upper chamber containing an uncoated (Corning 3422, USA) or Matrigel-coated insert (BD Biosciences 354480, USA) with 0.2 mL of serum-free medium to measure cell migration and invasion, respectively. The lower chambers were filled with DMEM containing 15% FBS. After 24 h, the cells located on the upper surface were removed using a cotton swab, and the cells on the lower surface were fixed in 4% polyoxymethylene and stained with crystal violet. In each well, the migratory or invading cells were counted and imaged in five randomly chosen fields at 10× magnification.

### Nude mouse liver metastasis tumour models

Male nude mice aged 4–6 weeks were purchased from the Shanghai Laboratory Animal Center of the Chinese Academy of Science (Shanghai, China). To establish the liver metastasis model, control or miR-1275-overexpressing MGC803 cells (2 × 10^6^) were resuspended in 0.05 mL of phosphate-buffered saline(PBS) and injected into the liver via the spleen. The mice were sacrificed after 1 month, at which time the livers were harvested, and the number of metastatic tumours in the liver was counted, and the expression levels of miR-1275 and JAZF1 in the liver was determined. All animals were sacrificed using transcardiac perfusion of ice-cold, heparinized phosphate buffered saline (PBS) followed by 4% paraformaldehyde (PFA).

### Chromatin immunoprecipitation (ChIP)

The ChIP assay was performed using a Magna ChIP A/G Chromatin Immunoprecipitation Kit (Millipore, USA). 1 × 10^7^ MGC803 cells were lysed with sodium dodecyl sulfate (SDS) lysis buffer and sonicated after the cross-link with 1% paraformaldehyde. The protein/DNA complexes were collected and immunoprecipitated with antibodies specific to JAZF1 (Abcam) or control goat IgG (Bioworld) and then reverse cross-linked to free DNA. The specific DNA fragments were quantitated by RT-PCR and normalized to the total input from the same cells.

### Western blot and IHC staining

*Western blot:* Cell lysates were analysed by Western blot using antibodies targeting JAZF1(Abcam), E-cadherin (Abcam), vimentin (Abcam), and GAPDH (Abcam). The protein bands were assessed using an Amersham Imager 600 (GE) as previously described [15].

#### IHC staining

IHC staining of patient tissue sections was performed as previously described. Tissues were fixed in 4% paraformaldehyde and sectioned at a thickness of 5 mm. After the sections were dewaxed with xylene and rehydrated with a graded series of ethanol, they were heated in an autoclave for three minutes with sodium citrate buffer (pH 6.0) and incubated with primary antibodies targeting E-cadherin (Abcam), anti-vimentin (Abcam), and matrix metalloproteinase 2 (MMP-2) (Abcam). Images viewed under a fluorescence microscopewere captured.

### Statistical analysis

SPSS 19.0 software was used to complete all statistical analyses. A paired Student’s t-test was used to comparing the mRNA levels of miR-1275 and JAZF1 between the paired tumour and nontumour tissues. The means of two groups were compared by an independent Student’s t-test. The association of miR-1275 expression with clinicopathological parameters were analysed by Pearson’s χ^2^ test. Kaplan-Meier curve were used for the survival analyses and log-rank tests for validation. Independent prognostic factors were identified by using univariate and multivariate Cox proportional hazard regression models.. Differences between groups were considered significant at *P* < 0.05. All data points represent the mean of three experiments.

## Results

### JAZF1 is a direct target of miR-1275 in GC cells

**Fig. 1:**
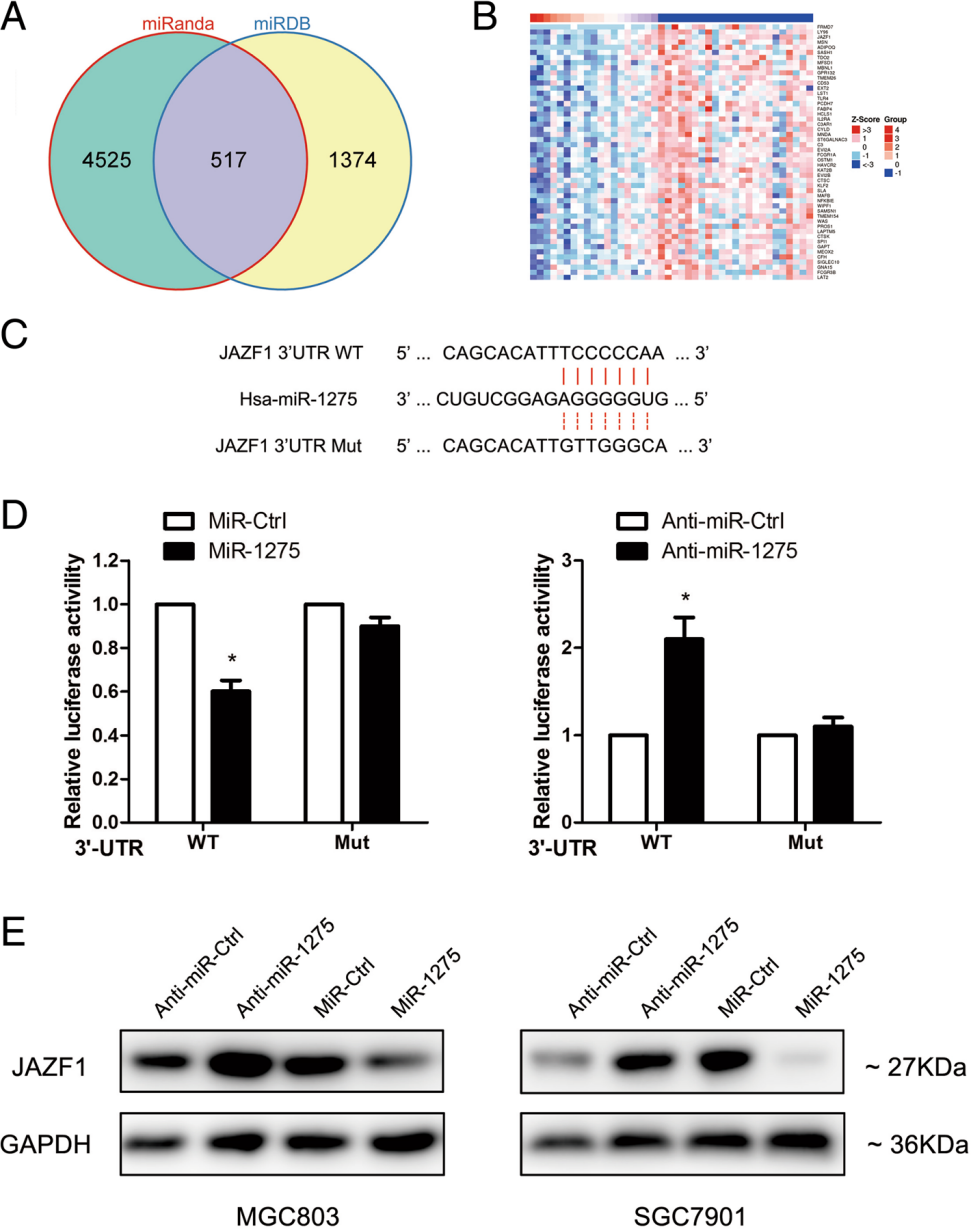
miR-1275 directly targets JAZF1 in GC cells. **a** Potential miR-1275 targets predicted by two computational prediction programs. **b** A portion of the cluster analysis of the mRNA expression profiles related to miR-1275 in the TCGA database. **c** Luciferase reporter plasmids were constructed as described in the Materials and Methods section. The sequences of the predicted miR-1275 binding sites within the 3’UTR of JAZF1 are shown, including the wild-type and mutant binding sites. **d** Relative luciferase activity was analysed after co-transfection of the above reporter plasmids or a mock reporter plasmid into 293 T cells infected with miR-1275 or anti-miR-1275. **e** Western blot analysis of GC cells transfected with miR-1275 or anti-miR-1275 as indicated

Using computational prediction programs (miRanda and TargetScan) and The Cancer Genome Atlas (TCGA) algorithms (Fig. 1a and b ), JAZF1 was identified as potentially regulated by miR-1275. To confirm this prediction, we then constructed a WT reporter and the corresponding mutant reporter (Fig. 1c). Ectopic miR-1275 overexpression effectively inhibited the luciferase activity of cells expressing the WT JAZF1–3’UTR. The luciferase activity was increased while miR-1275 was inhibited (Fig. 1d). Moreover, The effect of both miR-1275 and anti-miR-1275 were abolished when using the mutant reporter that the miR-1275 binding site in the 3’UTR of JAZF1 was replaced(Fig. 1d). To address the functional significance of miR-1275 binding to JAZF1 mRNA, we overexpressed miR-1275 in GC cells and found that JAZF1 expression was decreased at the translational level (Fig. 1e). These data therefore suggest that miR-1275 directly targets and negatively regulates transcripts of JAZF1 in GC cells.

### miR-1275 inhibits GC cell invasion and metastasis in vitro and in vivo by targeting JAZF1

**Fig. 2:**
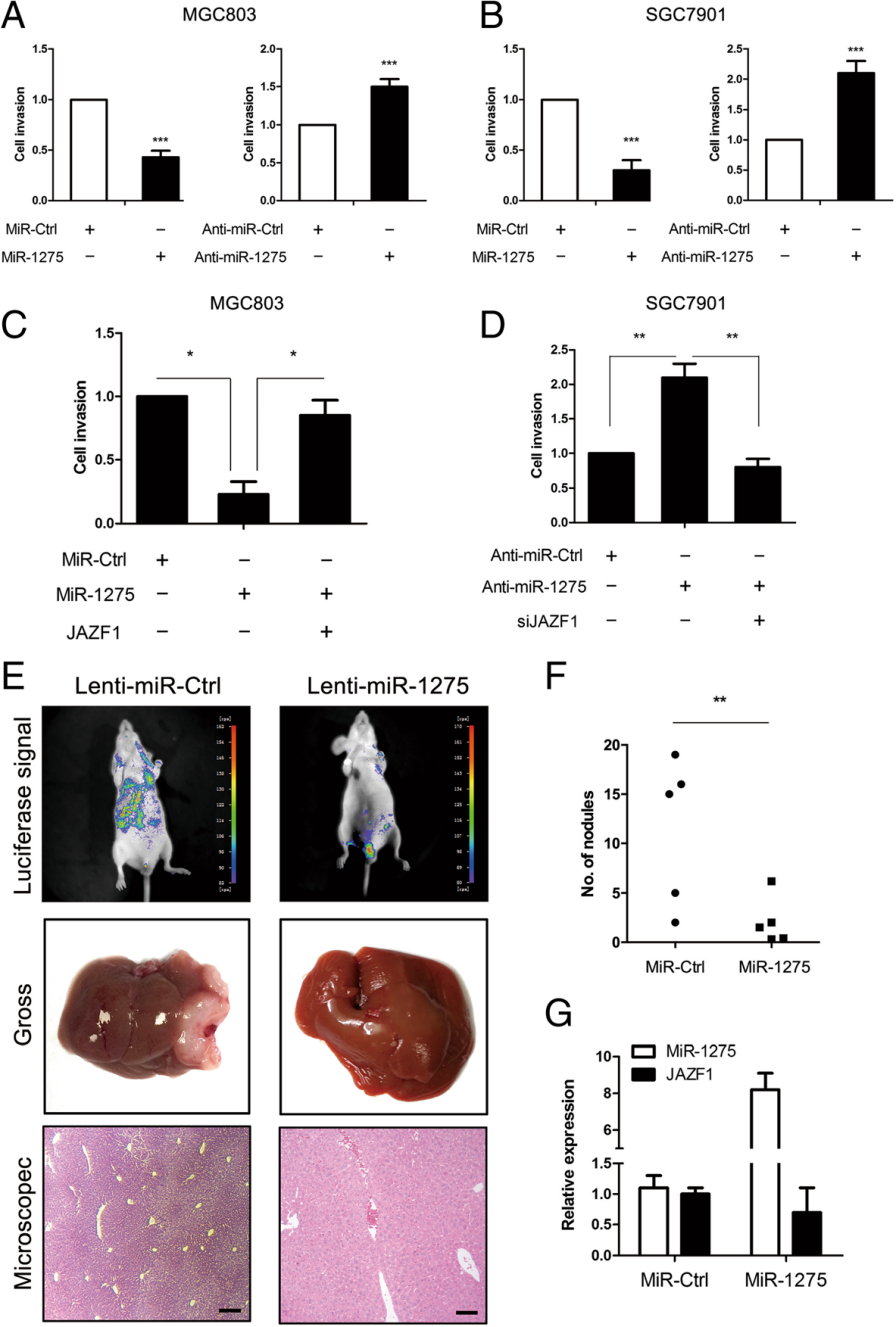
miR-1275 inhibits GC cell invasion in vitro and metastasis *in vivo*byregulating JAZF1. **a** and **b** MGC803 or SGC7901 cells transfected with miR-1275 and anti-miR-1275 were assayed for invasion capabilities. **c** Invasion assay of MGC803 cells transfected with miR-1275 and/or JAZF1 cDNA. **d** Invasion assay of SGC7901 cells transfected with anti-miR-1275 and/or siJAZF1. **e** and **f** Gross and microscopic examination for liver metastasis in both the control and miR-1275-overexpressing groups. **g** qRT-PCR analysis of JAZF1 expression in metastatic nodules in the livers of mice

To explore the potential role of miR-1275 in GC cells, we performed a transwell assay with a Matrigel-coated membrane. Ectopic expression of miR-1275 in cells was confirmed by qRT-PCR (Additional file 2: Figure S1A and B). Ectopic expression of miR-1275 inhibited the metastasis and invasion of GC cells, whereas GC cells treated with anti-miR-1275 were more invasive than the cells expressing miR-NC (Fig. 2a and b). Moreover, miR-1275 had no effect on the proliferation of GC cells (Additional file 2: Figure S1E and F), suggesting that the invasion inhibiting effect of miR-1275 was independent of cell proliferation. To determine whether JAZF1 was responsible for the miR-1275-dependent inhibition of GC cell metastasis indeed, we examined the effect of miR-1275 on GC cell invasion in the presence or absence of JAZF1 expression. As shown in Fig. 2c and Additional file 3: Figure S2B, exogenous JAZF1 expression reversed the majority of miR-1275-induced invasion. Meanwhile, the reciprocal experiment in SGC-7901 cells showed that JAZF1 silencing was partially blocked the invasion promotion effect of anti-miR-1275(Fig. 2d and Additional file 3: Figure S2A). To investigate the anti-GC effect of miR-1275 in vivo, we employed a liver tumour metastasis model via cecum injection of nude mice. As shown in Fig. 2e and f, the number of liver metastatic sites and the metastasis rate were significantly increased in mice injected with cells that overexpressed miR-1275. Moreover, the metastatic nodules produced by MGC803 cells stably expressing miR-1275 still presented lower levels of JAZF1 expression (Fig. 2g) than did nodules derived from the control cells. Collectively, our data demonstrated that miR-1275 plays a potential role in inhibiting metastasis and invasion in vitro and in vivo by targeting JAZF1.

### The regulatory effect of the molecular link of miR-1275 and JAZF1 on E-cadherin and vimentin expression in GC cells

**Fig. 3:**
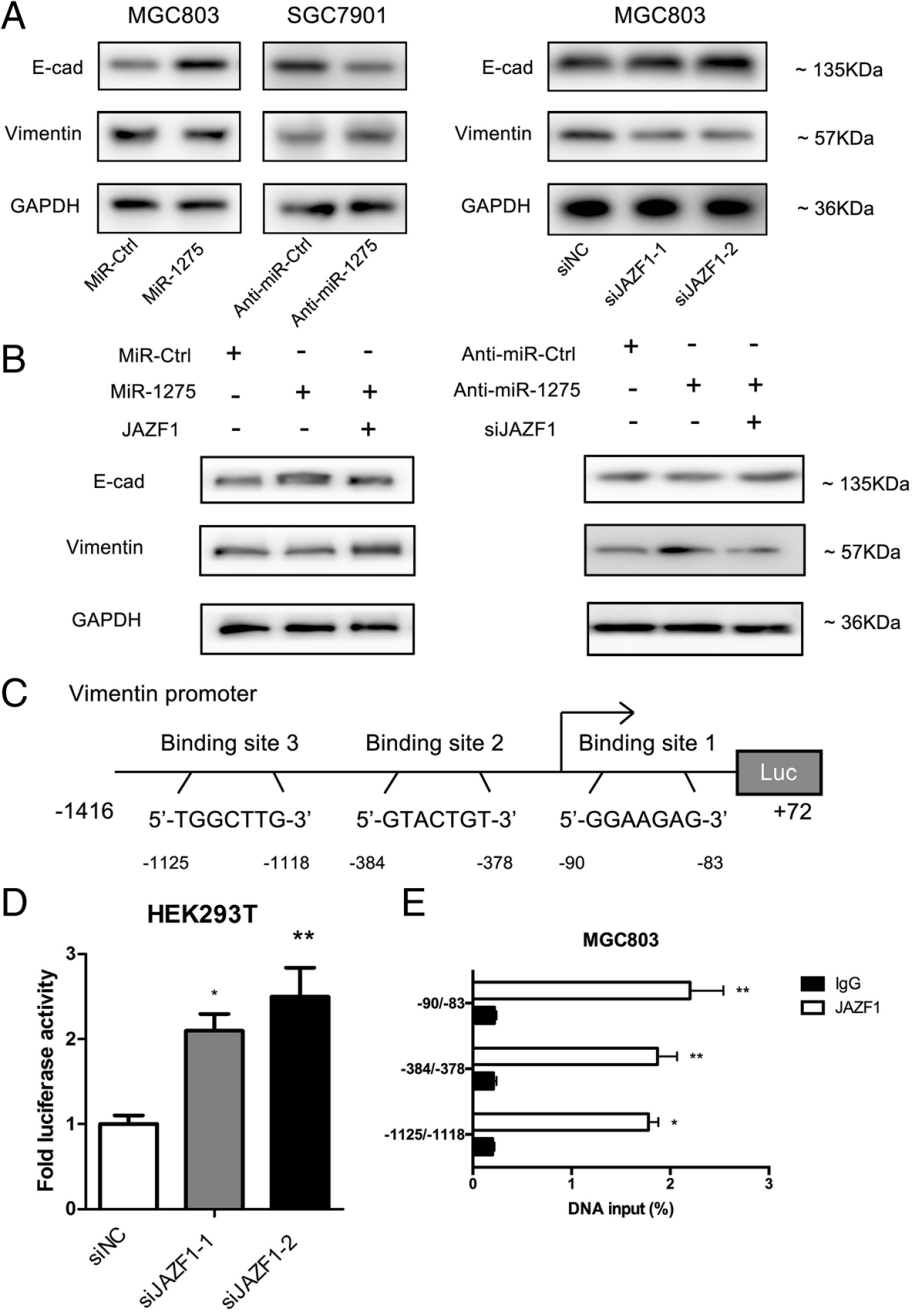
The miR-1275-JAZF1 link regulates vimentin and E-cadherin expression in GC cells. **a** Western blot analysis of vimentin and E-cadherin expression in MGC803 cells transfected with miR-1275 or miR-Ctrl and in SGC7901 cells transfected with anti-miR-1275 or anti-miR-Ctrl. Western blot analysis of E-cadherin and vimentin expression in MGC803 cells transfected with siJAZF1 or siNC. **b** GC cells were transfected with a JAZF1 expression vector or JAZF1 siRNA to restore or inhibit, respectively, the expression of E-cadherin and vimentin. **c** Schematic of the structure of the vimentin promoter. The numbers-1416 and + 72 depict the locations of the PCR primers used for vimentin promoter amplification. **d** Silencing JAZF1 decreased vimentin promoter activity. HEK293T cells were co-transfected with the vimentin-Luc reporter plasmid or pGL3 basic plasmid and Renilla with either siJAZF1 or siNC. Luciferase values were normalized to Renilla levels and expressed as the fold change over siNC. **e** ChIP analysis of JAZF1 at the vimentin promoter. Anti-JAZF1 or control IgG was used to immunoprecipitate the DNA-protein complex in MGC803 cells. Data regarding DNA precipitated by either anti-JAZF1 or control IgG are depicted as percentages of the total genomic DNA input. Numerical data are presented as the mean ± SD. **P < 0.01, ***P < 0.001

Based on the above data, the further mechanism of invasion and metastasis inhibition by miR-1275-JAZF1 in GC cells was subsequently investigated. Interestingly, Western blot analysis showed that miR-1275 overexpression significantly decreased the expression of vimentin and increased the expression of E-cadherin, whereas anti-miR-1275 showed the opposite trend (Fig. 3a and Additional file 3: Figure S2C). Moreover, the effect of JAZF1 on regulating EMT-related genes was analysed by Western blot. Correspondingly, knocking down JAZF1 led to E-cadherin expression increasing and vimentin expression decreasing(Fig. 3a and Additional file 3: Figure S2D). To examine gain and loss of JAZF1 function, a complementary approach experiment was performed. Specifically, we restored JAZF1 expression by vector in MGC803 cells and knocked down JAZF1 in SGC7901 cells. Restoration of JAZF1 expression recovered the expression of vimentin and E-cadherin by miR-1275-expressing MGC803 cells. Contrarily, knocking down JAZF1 expression abrogated the anti-miR-1275-mediatd inhibition of E-cadherin expression in SGC7901 cells (Fig. 3b). In addition, after analysing the vimentin promoter, we identified three potential JAZF1-binding sites in the vimentin promoter (Fig. 3c). Treatment with JAZF1 siRNA significantly decreased the activity at the vimentin promoter, indicating that the potential JAZF1-binding sites in the vimentin promoter were positive regulatory elements (Fig. 3c). Subsequent ChIP and qRT-PCR assays showed that compared with the control IgG immunoprecipitates, the JAZF1 immunoprecipitate enriched the potential binding sites (Fig. 3e). In general, these results indicated that the miR-1275-JAZF1 axis played a critical role in GC cell metastasis by regulating vimentin and E-cadherin expression.

### Correlations between miR-1275 expression and the clinicopathological characteristics and prognosis of GC

**Fig. 4:**
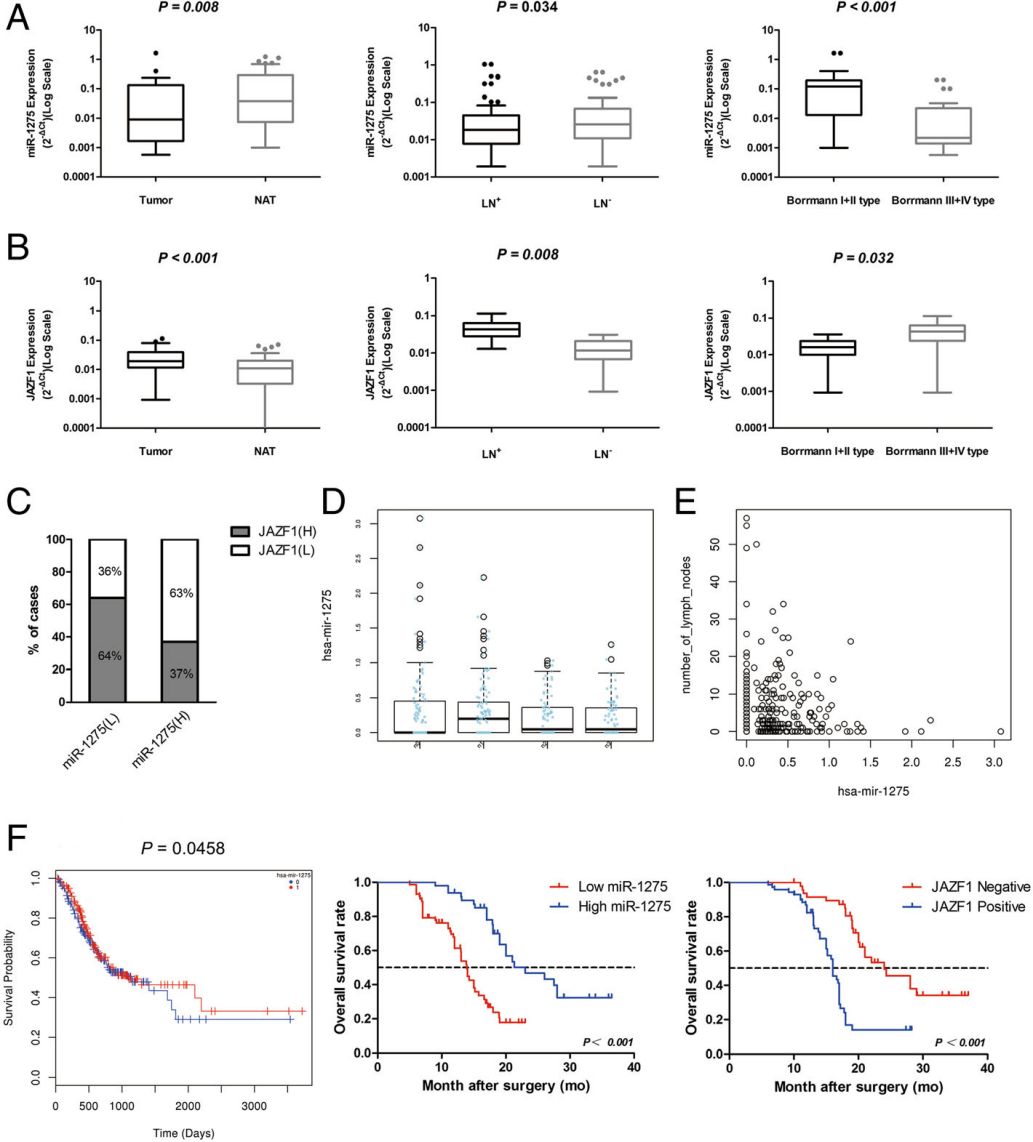
Low miR-1275 expression and high JAZF1 expression correlate with metastasis and poor survival in GC patients. **a** and **b**Expression of miR-1275 and JAZF1 in 120 pairs of samples from GC patients with and without metastasis and classified as either Borrmann type I + II or Borrmann type III + IV. The expression levels were determined by qRT-PCR and normalized to that of an endogenous control (U6 RNA). The data were analysed using the formula 2^-ΔCT^. **c** Inverse correlation of miR-1275 expression with JAZF1 mRNA expression in paired samples from 120 GC patients (*P* = 0.013, r = − 0.271, χ^2^ test). **d** and **e** qRT-PCR analysis of miR-1275 expression in different lymph node types from GC patient samples from the TCGA database. **f** Kaplan-Meier analysis of OS of GC patients according to the miR-1275 and JAZF1 mRNA expression profiles (left: TCGA database)

Based on the opposing functions of miR-1275 and JAZF1 in GC cell metastasis and invasion in vitro and in vivo, we analysed the correlation between miR-1275 and JAZF1 mRNA in 120 GC specimens from patients using qRT-PCR analysis. The expression of miR-1275 was reduced in GC tissues relative to that in adjacent nontumour tissues (*P* = 0.008) and negatively correlated with lymph node metastasis (*P* = 0.034) and Borrmann type (*P* < 0.001)(Table 1 and Fig. 4a). In contrast, JAZF1 expression was increased in GC tissues and was positively associated with lymph node metastasis (*P* = 0.008) and Borrmann type (*P* = 0.032)(Fig. 4b).

**Table 1:** Association of miR-1275 expression with the clinicopathological characteristics of GC

Variable	Category	Relative miR-1275 expression		χ2	*p*
		Low (69)	High (51)		
Age	< 60	18	18	1.184	0.277
	≥60	51	33		
Sex	Male	30	22	0.001	0.970
	Female	39	29		
Tumor location	Upper stomach	5	0	6.092	0.107
	Middle stomach	20	10		
	Lower stomach	40	36		
	Mixed	4	5		
*Borrmann type*	***Early stage***	***20***	***0***	***23.213***	***< 0.001***
	***I + II type***	***20***	***10***		
	***III + IV type***	***29***	***41***		
Histological differentiation	Well	10	5	0.610	0.737
	Moderate	21	17		
	Poor	38	29		
*Tumor invasion(AJCC)*	***Tis-T***_***2***_	***40***	***10***	***17.757***	***< 0.001***
	***T***_***3***_***-T***_***4***_	***29***	***41***		
*Lymph node metastasis*	***Yes***	***31***	***48***	***31.546***	***< 0.001***
	***No***	***38***	***3***		
*TNM stage(AJCC)*	***I-II***	***39***	***10***	***16.540***	***< 0.001***
	***III-IV***	***30***	***41***		

Bold values are significant, *P* < 0.05

As expected, JAZF1, a target gene of miR-1275, was inversely correlated with miR-1275 expression on mRNA level (Spearman r = − 0.271, *P* = 0.013) (Fig. 4c).

More intriguingly, the survival analysis showed better OS in GC patients with higher miR-1275 expression and lower JAZF1 expression than in patients with lower miR-1275 expression (1- and 3-year OS: 55.7 and 33.5% vs. 34.0 and 17.0%, *P* = 0.0013) and higher JAZF1 expression (1- and 3-year OS: 72.3 and 36.6% vs. 17.7 and 17.7%, *P* < 0.001) (Fig. 4f). These results were confirmed by univariate and multivariate survival analyses using the TCGA database (Table 2 and Fig. 4d and e). These data reveal that the inverse relationship of miR-1275 and JAZF1 is associated with survival and GC metastasis.

**Table 2:** Univariate and multivariate analyses of clinical variables contributing to overall survival

Variable	Univariate analysis		Multivariate analysis	
	HR (95%CI)	*p*	HR (95%CI)	*p*
Age (< 60 vs. ≥60)	0.950 (0.568–1.590)	0.846	–	–
Sex (male vs. female)	0.863 (0.372–2.001)	0.731	–	–
Tumor location (Upper or middle vs. Lower stomach)	1.378 (0.474–4.006)	0.556	–	–
Histological differentiation (well or moderate vs. poor)	0.634 (0.329–1.224)	0.175	–	–
Tumor invasion (AJCC) (Tis-T2 vs. T3-T4)	1.512 (0.617–3.708)	0.366	–	–
*Lymph node metastasis (yes* vs. *no)*	***1.359 (0.592–3.122)***	***0.043***	1.245 (0.519–2.983)	0.623
*TNM stage (AJCC) (I-II*vs.*III-IV)*	***1.477 (0.548–3.981)***	***0.002***	1.363 (0.480–3.874)	0.561
Surgery type (curative resection vs. palliative)	1.205 (0.521–2.786)	0.663	–	–
*miR-1275 expression in tumor (low vs. high)*	***0.268(0.154–0.464)***	***0.001***	***0.126 (0.035–0.448)***	***0.001***

Bolded values are significant, *P* < 0.05; *CI*, Confidence interval; *HR*, Hazard ratio

### High JAZF1 protein expression is associated with low E-cadherin and high vimentin protein expression in GC tissues

**Fig. 5:**
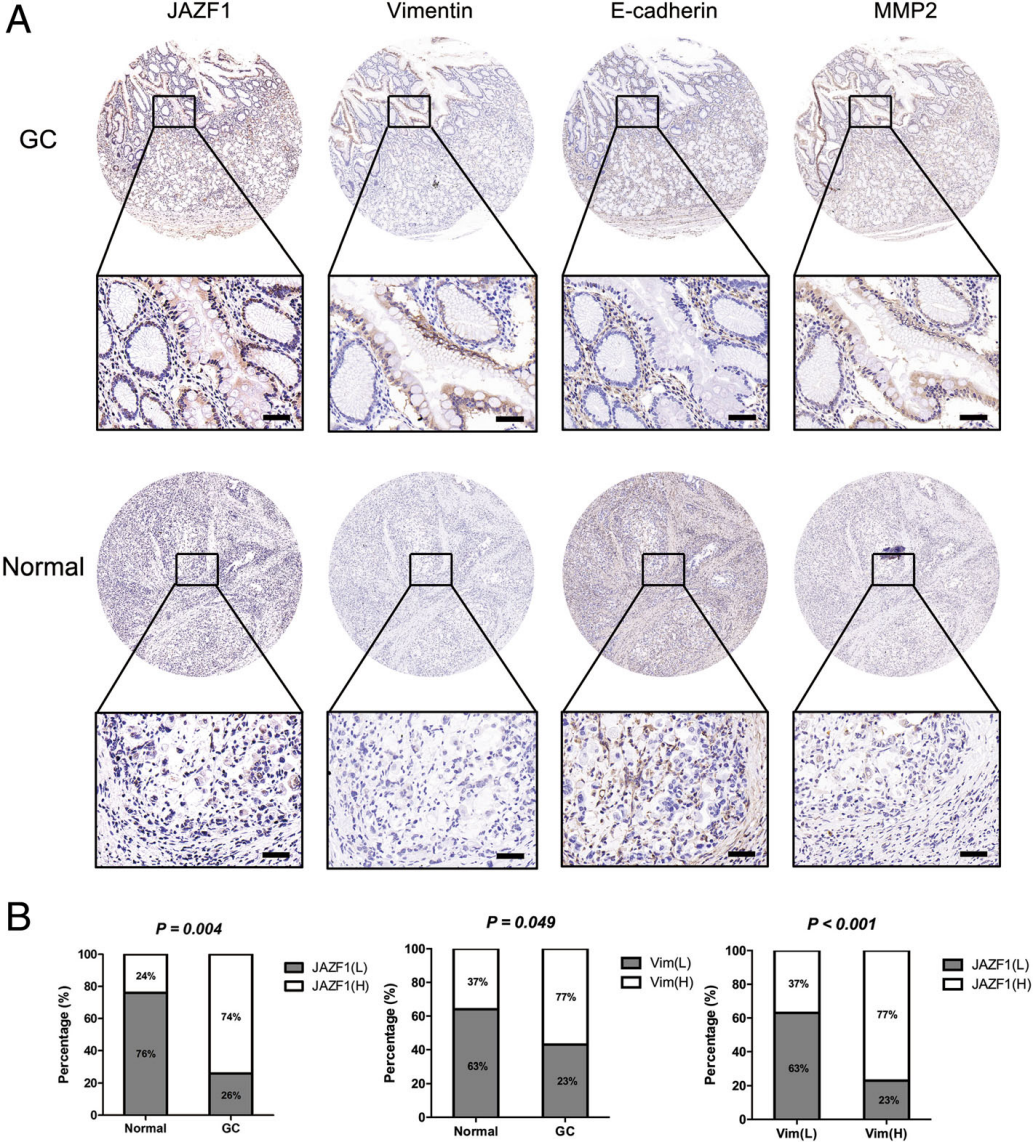
JAZF1 expression is inversely associated with E-cadherin expression and positively associated with vimentin expression. Four tissue microarrays of consecutive immunostained sections underwent specific antibodies against JAZF1, vimentin, E-cadherin and MMP-2. **a** Representative images of JAZF1, vimentin, E-cadherin and MMP-2 protein expression levels in healthy and cancerous specimens (magnification: × 400 for the inserts, × 50 for all others). **b** JAZF1 and vimentin expression levels were significantly higher in GC tissues than in healthy gastric tissues(*P* = 0.006), whereas E-cadherin expression was significantly higher in healthy gastric tissues than in GC tissues. JAZF1 expression was positively correlated with vimentin expression (*P* < 0.001, *r* = − 0.400)

Immunohistochemistry were performed in 120 pairs of GC and healthy gastric tissues for JAZF1, vimentin, E-cadherin and MMP-2 (Fig. 5a) to validate the regulatory relationship among JAZF1, E-cadherin and vimentin. In GC tissues, the JAZF1, vimentin and MMP-2 protein expression levels were higher, and the E-cadherin protein expression level was lower than the corresponding levels in normal tissues (Fig. 5a and Additional File 4: Figure S3). Of note, JAZF1 had a significant correlation with vimentin and inversely with E-cadherin in GC tissues (Fig. 5b). The above data suggest that high vimentin expression and low E-cadherin expression had an notablely relationship with the high expression of JAZF1 in the progression of GC.

## Discussion

Previous evidence has indicated that miRNAs play crucial roles in GC, including miR-17 [25], miR-100 [26], miR-125b [27], miR-133b [28] and miR-145 [29],and miRNAs are novel candidates in the promotion GC metastasis with definite molecular mechanisms that require further exploration. In this study, data from both in vitro GC cell tests and the analysis of tissue from GC patients indicate that miR-1275 inhibited GC metastasis. Our in vitro results from 2 GC cell lines demonstrate that miR-1275 directly targeted JAZF1 to inhibit metastasis both in vitro and in vivo in GC cells and that vimentin and E-cadherin were direct targets of JAZF1. Our results also showed that high levels of JAZF1 protein expression were associated with high vimentin and low E-cadherin protein expression in GC specimens. Therefore, the miR-1275-JAZF1-vimentin/E-cadherin axis, in which miR-1275 inhibits vimentin and increases E-cadherin expression by targeting JAZF1, is a likely contributor to the development of GC.

miR-1275 is an intergenic miRNA encoded by chromosome 6. It was suggested that miR-1275 acts as a tumour suppressor in several types of cancers [30–33], and miR-1275 overexpression has been reported to inhibit malignant cell behaviours and proliferation in hepatocellular carcinoma simultaneously by regulating oncogenic insulin-like growth factor-2 [30–32]. Although miR-1275 has been identified to be associated with GC, the mechanism of miR-1275 in gastric tumorigenesis needs to be investigated.

Several studies have demonstrated that JAZF1 is related to a diversity of diseases, including diabetes, cardiac disease, prostate cancer and endometrial stromal sarcoma [22, 34–36]. JAZF1 as a transcriptional repressor of TR4, previous studies focused on JAZF1 function in gluconeogenesis regulation and diabetes [17, 36, 37]. Additionally, JAZF1 functions in malignancies, especially endometrial stromal tumours and prostate cancer [22–24]. Rearrangement of JAZF1-SUZ12 in endometrial stromal tumours exacerbates cancer by repressing the PRC2 complex to inhibit histone methyltransferase [22]. Here, we demonstrate the novel miRNA-mediated downregulation of JAZF1.

In our study, miR-1275 expression was found frequently decreased in GC tumour tissues, suggesting that miR-1275 might play an inhibitory role in the GC developing process. Importantly, immunohistochemistry showed that low miR-1275expression was negatively correlated with the Borrmann type, lymph node metastasis, and OS of GC in our cohort of120 patients. In addition, miR-1275 was identified as a independent factor of poor prognosis of GC by Cox proportional hazard regression analysis. Low miR-1275 expression may be used for GC early detection and more accurate prognosis.

New target genes of JAZF1 need to be discovered. However, the mechanism by which JAZF1 regulates GC metastasis remains to be elucidated. Epithelial-to-mesenchymal transition (EMT) is the transition of cells from a proliferative epithelial phenotypeto a migratory and invasive mesenchymal phenotype. During EMT progression, GC cells lose their polarity and their capacity for adhesion, which weakens intercellular connections [38]. It has been proven in many tumours that vimentin plays an important role in tumour invasion and metastasis [38–40], and vimentin promotes GC progression by repressing E-cadherin. Previous studies have shown that JAZF1 promotes Slug expression [24], and because Slug expression significantly increases vimentin expression, this suggests an indirect means by which JAZF1 can regulate vimentin. Now, we demonstrate that JAZF1 can act as a transcriptional activator and bind to the vimentin promoter directly to positively regulate its expression. Therefore, JAZF1 could promote GC tumour metastasis by promoting vimentin expression and suppressing E-cadherin expression via direct transcriptional repression.

## Conclusion

In summary, our finding revealed the role of the miR-1275-JAZF1-vimentin/E-cadherin axis in GC metastasis. The results that decreased miR-1275 expression suppresses GC metastasis by regulating vimentin/E-cadherin via direct targeting of JAZF1 highlights the potential of miR-1275 as a novel target in GC metastasis.

## Additional files

Additional file 1: **Table S1.** Patient Demographics and Clinical Characteristics. (DOCX 16 kb)

Additional file 2: **Figure S1.** miR-1275has no effect on proliferation of GC cell. (A-D) The confirmed transfection efficiency of miR-1275 or anti-miR-1275 and si-JAZF1 or JAZF1 overexpression in MGC803 and SGC7901 cells. RT-qPCR was performed to analyze the transfection efficiency of miR-1275, anti-miR-1275, si-JAZF1 or JAZF1. (E-F) CCK8 proliferation assay of miR-Ctrl and miR-1275 or anti-miR-1275. (JPG 1607 kb)

Additional file 3: **Figure S2.** MiR-1275 inhibits GC cell invasion in vitro through regulation of EMT. (A) Invasion assay of SGC7901 cells transfected with miR-1275 and/or JAZF1 cDNA. (B) Invasion assay of MGC803 cells transfected with anti-miR-1275 and/or siJAZF1. (C) Western blot analysis of Vimentin and E-cad expression in SGC7901 cells transfected with miR-1275 or miR-Ctrl and in MGC803 cells transfected with anti-miR-1275 or anti-miR-Ctrl. (JPG 1287 kb)

Additional file 4: **Figure S3.** MMP2 expression levels were significantly higher in GC tissue species than that in the normal gastric tissue species (*P* = 0.049), whereas E-cad expression level was significantly higher in the normal gastric tissue species than those in the GC tissue species(*P* < 0.01). (JPG 997 kb)

Additional file 5: **Table S2.** The nucleotides applied in the study. (DOCX 15 kb)

## Data Availability

The datasets used and/or analyzed during the current study are available from the corresponding author upon reasonable request.

## Abbreviations

AJCC: American Joint Committee on Cancer
Anti-miR-1275: miR-1275 inhibitor
Anti-NC: microRNA inhibitor control
ChIP: Chromatin immunoprecipitation
EMT: Epithelial to mesenchymal transition
GBC: Gallbladder cancer
GC: Gastric cancer
IHC: Immunohistochemistry
JAZF1: Juxtaposed with another zinc finger protein 1
miR-1275: microRNA-1275
miRNA(s): microRNA(s)
MMP-2: Matrix metalloproteinase-2
mRNA(s): messenger RNA(s)
Mut: mutant type
NAT(s): Adjacent nontumorous tissues
OS: Overall survival
qPCR: quantitative real-time PCR
siRNAs: small interfering RNAs
TCGA: The Cancer Genome Atlas
TR4: Testicular nuclear receptor 4
UTR: Untranslated region
WT: Wild type
