# Supplementary material for: MicroRNA-1275 inhibits cell migration and invasion in gastric cancer by regulating vimentin and E-cadherin via JAZF1
Source: BMC Cancer. 2019 Jul 29;19:740. doi: 10.1186/s12885-019-5929-1 (PMC6664777; doi:10.1186/s12885-019-5929-1)
Supplement: Supplementary file 1 — Table S1. Patient Demographics and Clinical Characteristics. (DOCX 16 kb) [file 12885_2019_5929_MOESM1_ESM.docx]

| **Supplementary Table 1 Patient Demographics and Clinical Characteristics** | | |
| --- | --- | --- |
| Characteristic |  | Number of Patients |
| Patients |  | 120 |
| male |  | 42 |
| female |  | 58 |
| Age (years) |  | 44-84, median=67 |
| Tumor size (cm) |  | 0.8-9.0, median=2.75 |
| Histology differentiation | |  |
| Well |  | 10 |
| Moderate |  | 28 |
| Poor |  | 82 |
| Borrmann type |  |  |
| Early stage |  | 20 |
| I+II type |  | 30 |
| III+IV type |  | 70 |
| Local invasion |  |  |
| T_3_-T_4_ |  | 80 |
| Lymph node metastasis | |  |
| Yes |  | 79 |
| No |  | 41 |
| TNM stage |  |  |
| I-II |  | 70 |
| III-IV |  | 50 |
| Time of follow-up (months) | | 1-36, median=14.3 |
| TNM, tumor-nodes-metastasis, based on the American Joint Committee on Cancer/International Union Against Cancer staging manual (7th edition, 2009) | | |
|  |  |  |
