# Supplementary figures and images for: MicroRNA-1275 inhibits cell migration and invasion in gastric cancer by regulating vimentin and E-cadherin via JAZF1
Source: BMC Cancer. 2019 Jul 29;19:740. doi: 10.1186/s12885-019-5929-1 (PMC6664777; doi:10.1186/s12885-019-5929-1)

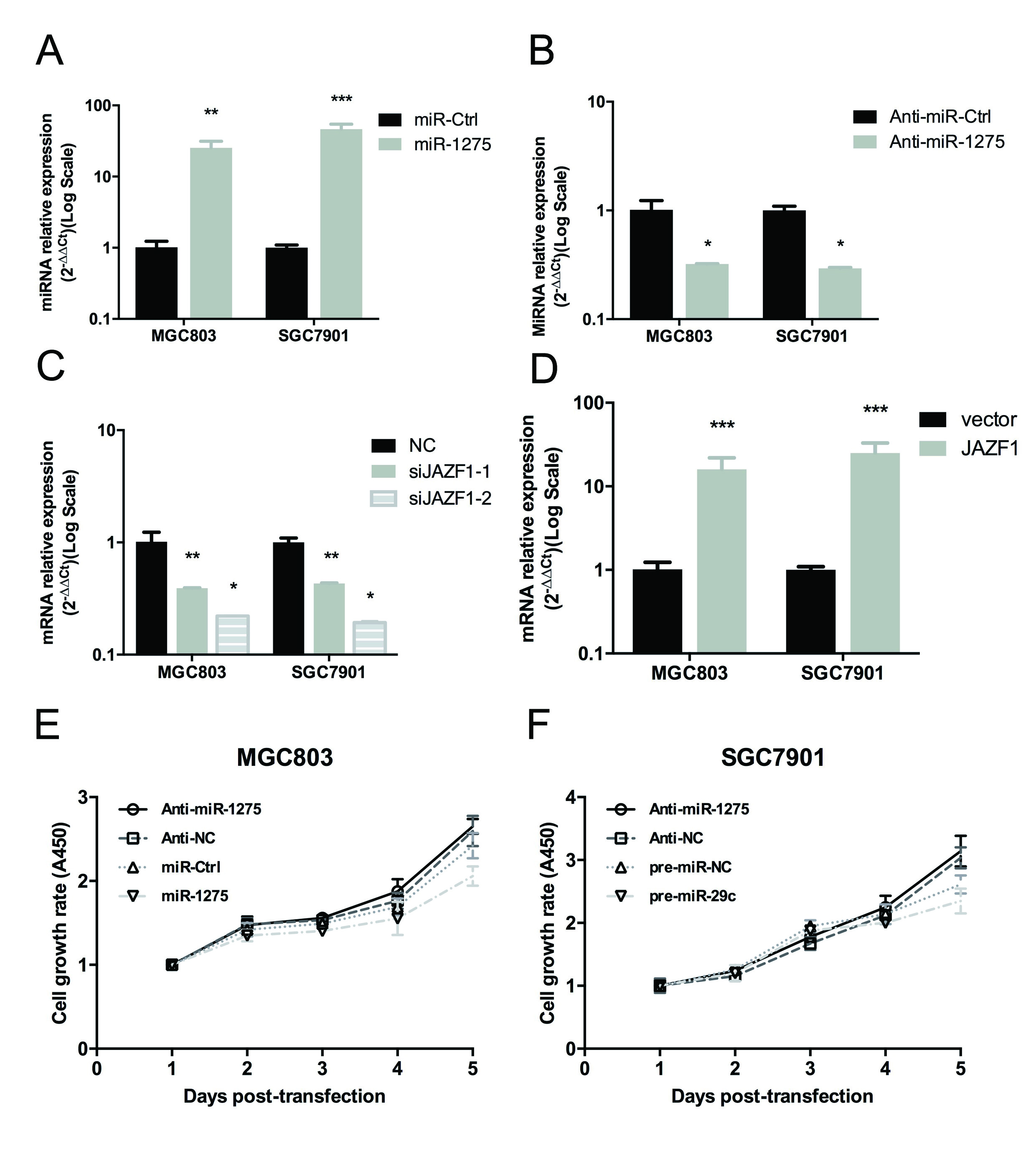

Supplement: Supplementary file 2 — Figure S1. miR-1275has no effect on proliferation of GC cell. (A-D) The confirmed transfection efficiency of miR-1275 or anti-miR-1275 and si-JAZF1 or JAZF1 overexpression in MGC803 and SGC7901 cells. RT-qPCR was performed to analyze the transfection efficiency of miR-1275, anti-miR-1275, si-JAZF1 or JAZF1. (E-F) CCK8 proliferation assay of miR-Ctrl and miR-1275 or anti-miR-1275. (JPG 1607 kb) [file 12885_2019_5929_MOESM2_ESM.jpg]

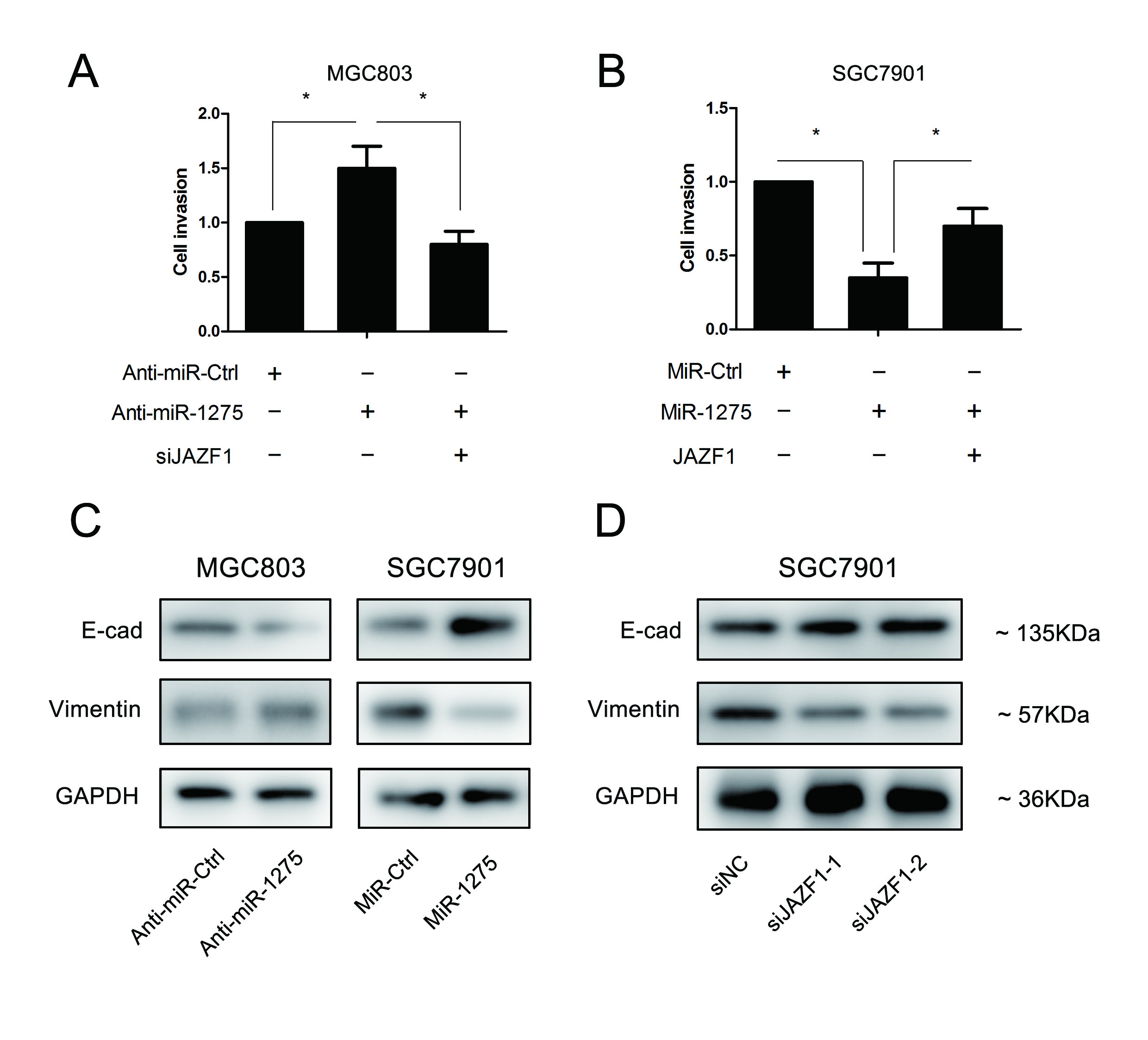

Supplement: Supplementary file 3 — Figure S2. MiR-1275 inhibits GC cell invasion in vitro through regulation of EMT. (A) Invasion assay of SGC7901 cells transfected with miR-1275 and/or JAZF1 cDNA. (B) Invasion assay of MGC803 cells transfected with anti-miR-1275 and/or siJAZF1. (C) Western blot analysis of Vimentin and E-cad expression in SGC7901 cells transfected with miR-1275 or miR-Ctrl and in MGC803 cells transfected with anti-miR-1275 or anti-miR-Ctrl. (JPG 1287 kb) [file 12885_2019_5929_MOESM3_ESM.jpg]

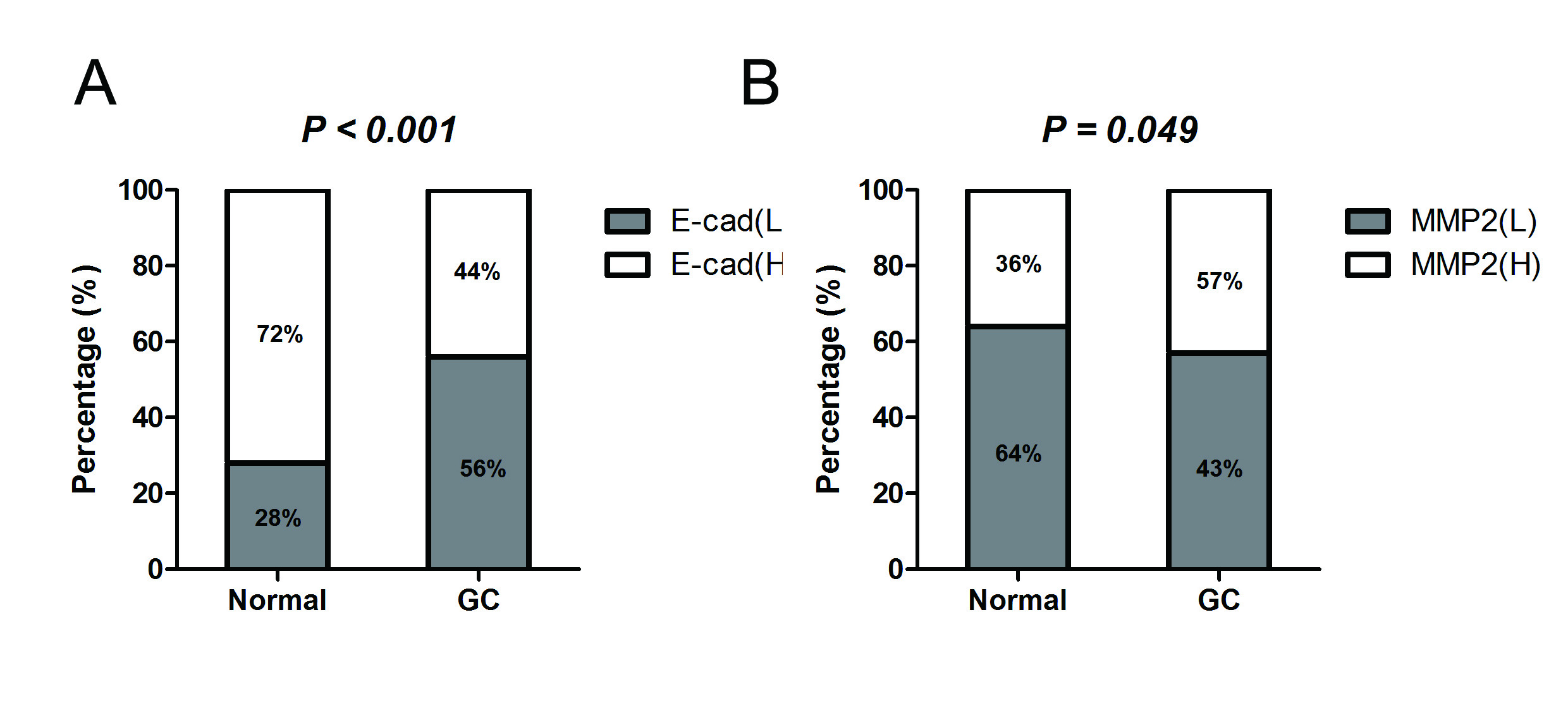

Supplement: Supplementary file 4 — Figure S3. MMP2 expression levels were significantly higher in GC tissue species than that in the normal gastric tissue species (P = 0.049), whereas E-cad expression level was significantly higher in the normal gastric tissue species than those in the GC tissue species(P < 0.01). (JPG 997 kb) [file 12885_2019_5929_MOESM4_ESM.jpg]
