# Supplementary material for: MicroRNA-1275 inhibits cell migration and invasion in gastric cancer by regulating vimentin and E-cadherin via JAZF1
Source: BMC Cancer. 2019 Jul 29;19:740. doi: 10.1186/s12885-019-5929-1 (PMC6664777; doi:10.1186/s12885-019-5929-1)
Supplement: Supplementary file 5 — Table S2. The nucleotides applied in the study. (DOCX 15 kb) [file 12885_2019_5929_MOESM5_ESM.docx]

| **Supplementary Table 2. The nucleotides applied in the study** | | |
| --- | --- | --- |
| Description | Name | Sequence |
| Primers for qRT-PCR | hsa-miR-1275 | CGGTAGCACCATTTGAAATCGGTTA |
|  | JAZF1-F | GGAGTCGGACAGCGATGATGAGT |
|  | JAZF1-R | GCTTCTCTTCCCCTCCATTCA |
|  | GAPDH-F | AGAAGGCTGGGGCTCATTTG |
|  | GADPH-R | AGGGGCCATCCACAGTCTTC |
| siRNA for JAZF1 | siRNA-1 | 5'-UCUGUGACCAUUCUUAGCGUG-3' |
|  | siRNA-2 | 5'-UUCACAUUCUUGUAUCUCUUU-3' |
| Primers for chip analysis | Vimentin(-90/-83) | F:GAGTTTCCTCTTTCACCCAG |
|  |  | R:CTCCCAGATCACGATTGCA |
|  | Vimentin(-384/-378) | F:CAGGCTTTAGCGAGTTAT |
|  |  | R:CTCAGTGCTACCAACTTACA |
|  | Vimentin(-1125/-1118) | F:TTGCACAGGGTGGAGTG |
|  |  | R:AGCCAGGCGTGATGGT |
